# Nanosecond Laser-Textured Copper Surfaces Hydrophobized with Self-Assembled Monolayers for Enhanced Pool Boiling Heat Transfer

**DOI:** 10.3390/nano12224032

**Published:** 2022-11-16

**Authors:** Matic Može, Matevž Zupančič, Miha Steinbücher, Iztok Golobič, Henrik Gjerkeš

**Affiliations:** 1 Faculty of Mechanical Engineering, University of Ljubljana, Aškerčeva 6, 1000 Ljubljana, Slovenia; 2 Helios TBLUS d.o.o., Količevo 65, 1230 Domžale, Slovenia; 3 School of Engineering and Management, University of Nova Gorica, Vipavska 13, 5000 Nova Gorica, Slovenia

**Keywords:** nucleate boiling, heat transfer enhancement, heat transfer coefficient, surface functionalization, laser texturing, hydrophobization

## Abstract

Increased cooling requirements of many compact systems involving high heat fluxes demand the development of high-performance cooling techniques including immersion cooling utilizing pool boiling. This study presents the functionalization of copper surfaces to create interfaces for enhanced pool boiling heat transfer. Three types of surface structures including a crosshatch pattern, shallow channels and deep channels were developed using nanosecond laser texturing to modify the surface micro- and nanomorphology. Each type of surface structure was tested in the as-prepared superhydrophilic state and superhydrophobic state following hydrophobization, achieved through the application of a nanoscale self-assembled monolayer of a fluorinated silane. Boiling performance evaluation was conducted through three consecutive runs under saturated conditions at atmospheric pressure utilizing water as the coolant. All functionalized surfaces exhibited enhanced boiling heat transfer performance in comparison with an untreated reference. The highest critical heat flux of 1697 kW m^−2^ was achieved on the hydrophobized surface with shallow channels. The highest heat transfer coefficient of 291.4 kW m^−2^ K^−1^ was recorded on the hydrophobized surface with deep channels at CHF incipience, which represents a 775% enhancement over the highest values recorded on the untreated reference. Surface microstructure was identified as the key reason for enhanced heat transfer parameters. Despite large differences in surface wettability, hydrophobized surfaces exhibited comparable (or even higher) CHF values in comparison with their hydrophilic counterparts, which are traditionally considered as more favorable for achieving high CHF values. A significant reduction in bubble departure diameter was observed on the hydrophobized surface with deep channels and is attributed to effective vapor entrapment, which is pointed out as a major contributing reason behind the observed extreme boiling heat transfer performance.

## 1. Introduction

### 1.1. Boiling Heat Transfer

Boiling represents an efficient heat transfer mechanism allowing the removal of concentrated thermal loads at small temperature differences between the hot surface and the cooling medium. Its uses include cooling of fuel rods and reactor pressure vessels in certain types of nuclear reactors [1,2] and heat removal from (micro)electronic processors and components [3,4]. The main heat transfer parameters that define its heat transfer intensity are the heat flux (i.e., the heat transfer rate per unit area), surface superheat (i.e., temperature difference between the boiling surface and the cooling medium) and the heat transfer coefficient (i.e., the ratio between heat flux and surface superheat in a given operating point). It is desirable for boiling to be able to remove large heat fluxes at low surface superheat, which results in high values of the heat transfer coefficient (HTC).

Pool boiling is the basic form of boiling, taking place in a macroscopically stationary pool of liquid. Furthermore, pool boiling can be classified into several regimes based on the physical phenomena on the boiling surface. The most important regime from an engineering standpoint is nucleate boiling, which starts when the first vapor bubbles start to form on the surface and natural convection is no longer intense enough to remove the heat load. This point is denoted as onset of nucleate boiling (ONB). With increasing heat flux, the number of active nucleation sites producing bubbles and bubble departure frequency increase, alongside increasing coalescence (i.e., merging) of bubbles. When the coalescence is intense enough to cause a vapor film to form on the surface, the so-called critical heat flux (CHF) is said to have been reached and the process transitions towards film boiling. CHF incipience therefore represents the upper heat flux limit of the nucleate boiling regime, which should not be exceeded during operation to avoid a massive increase in surface temperature and damage to or destruction of the cooled component.

While the boiling offers intense heat transfer with heat transfer coefficients for pool boiling of saturated water at atmospheric pressure reaching several tens of kW m^−2^ K^−1^ on polished or otherwise untreated surfaces, several applications require the enhancement of boiling intensity in the form of earlier ONB, increased HTC and delayed CHF, therefore widening the range within which efficient and safe cooling can be provided. The condition of the boiling surface and its interaction with the working fluid (namely wettability and wickability) importantly influence the intensity of boiling heat transfer and have been focused upon by researchers trying to improve phase-change cooling in the last decade. Based on the current knowledge, the boiling surface should include cavities or other morphological features with the ability to entrap vapor and therefore serve as active nucleation sites. Additionally, higher surface wettability (e.g., (super)hydrophilicity) favorably affects the delay of CHF, but at the cost of delayed boiling onset. Conversely, poorly wettable (hydrophobic) surfaces expedite the ONB but are typically more inclined towards early vapor film formation, thus lowering the CHF value. Nevertheless, several studies have shown that correct degassing of otherwise superhydrophobic textured surfaces can simultaneously allow the enhancement of all the aspects of nucleate boiling; that is, lowering the ONB and increasing both the HTC and the CHF [5,6].

### 1.2. Boiling Heat Transfer Enhancement via Surface Functionalization

Several recent reviews [7,8,9,10,11] revealed the vast number of approaches to boiling heat transfer enhancement. These include the use of surface treatments to induce surface micro- and nanostructure, porous coatings, hydrophilic or hydrophobic coatings, deposition of nanoparticles and/or use of nanofluids, machining of extended surfaces or fabrication of various surface features on the (sub)micron scale. Some recent studies are reviewed in the following paragraphs.

Ahmadi et al. [12] studied the effect of functional surfaces with gradient mixed wettability on flow boiling in a high aspect ratio microchannel. They found a notable heat transfer enhancement using biphilic surfaces with mixed wettability regions compared to the reference sample. Visualization showed that biphilic surfaces reduce the bubbly flow regime and extend the slug regime. Le Ba et al. [13] added halloysite nanotubes to deionized water to create a nanofluid, which was boiled on horizontal copper tubes. A minor heat transfer enhancement was obtained at low heat flux values. Cho et al. [14] used powder injection molding to fabricate micro-patterned surfaces with submillimeter pins. Two types of functionalized surfaces were prepared, and a CHF and HTC enhancement was recorded on both of them compared to the entreated reference surface. Može et al. [15] investigated the difference in boiling on a hydrophilic and a hydrophobic surface with the same morphology by first performing boiling tests on a titanium surface (functionalized through hydrothermal treatment to induce TiO_2_ nanotube growth) in the as-fabricated state, followed by drying, hydrophobization, and further tests in the hydrophobic state. It was shown that hydrophobization importantly decreases the ONB, increases the HTC and significantly narrows the surface temperature distributions. Freitas et al. [16] studied boiling of nanofluids on biphilic surfaces, both experimentally and numerically. It was found that the biphilic pattern importantly influences bubble coalescence, while the nanofluid had only a minor effect on the boiling performance. Galicia et al. [17] investigated the enhancement of subcooled flow boiling using a high porosity sintered fiber coating on the surface. The results showed that while the porous coating can enhance the heat transfer, it will degrade the performance if it is too thick. The enhancement was attributed to increased nucleation site density, larger surface area, enhanced water supply ability through the porous structure, and the vapor entrapment ability of the porous coating. Similar results were obtained in a related study by Otomo et al. [18]. Može et al. [19] used chemical etching to fabricate microcavities or porous structures on aluminum samples, while selected samples were subsequently also hydrophobized. Pool boiling experiments with water and self-rewetting fluids showed that major enhancements of the heat transfer coefficients are possible with its highest recorded values exceeding 300 kW m^−2^ K^−1^. Kaniowski and Pastuszko [20] investigated the effect of open microchannels, fabricated through machining, on pool boiling heat transfer with water. The obtained results revealed that major enhancement of both the CHF and the HTC are possible, with the highest reported HTC reaching almost 400 kW m^−2^ K^−1^. Kamel and Lezsovits [21] used a tungsten oxide nanofluid to enhance pool boiling performance, but the results showed a decrease of the HTC in most cases. The authors attributed this to deposition of a nanoparticle layer onto the boiling surface, which increased the thermal resistance. Kim et al. [22] fabricated a microdome structure on an aluminum substrate using direct metal forming and a custom glassy carbon mold. A moderate increase in both the HTC and CHF was observed during experiments with pool boiling of water. Može et al. [23] used two types of chemical treatment to induce nanostructure formation on copper surfaces, which were subsequently tested under pool boiling conditions with water, water/butanol mixtures and Novec 649 dielectric fluid. Both types of functionalized surfaces offered enhanced heat transfer, while a degradation of surface was detected when the butanol/water self-rewetting mixture was boiled on them, which was attributed to a chemical reaction with copper compounds on the surface. Mukherjee et al. [24] investigated the possibility of enhancing pool boiling heat transfer with silica nanoparticles mixed into water to create a non-metallic-oxide nanofluid. While a modest improvement of the HTC and CHF was observed, deposition of nanoparticles onto the surface during boiling resulted in increased bubble diameter and reduced active nucleation site density. Pranoto et al. [25] studied the role of pin fin array configurations on pool boiling heat transfer enhancement and found that the pin fin arrays importantly influence bubble dynamics and affect the boiling performance. Hadžić et al. [26] showed that laser-textured surfaces, already enhancing the boiling performance on their own, can be combined with boiling of a nanofluid to further enhance the CHF value. However, this enhancement is at the expense of significantly increased surface superheat, resulting in lower HTC values. Orman et al. [27] researched the influence of surface laser treatment on boiling heat transfer using water and ethanol. A significant influence of laser processing parameters on heat flux dissipated from surfaces with laser-induced grooves or microfins was observed. Recently, Sajjad et al. [28] used a deep learning approach coupled with explainable artificial intelligence to evaluate the liquid-to-vapor phase change heat transfer in nanoporous surface coatings alongside a parameter sensitivity analysis of nanoporous surface coatings. Heat flux, the substrate’s thermal conductivity, and pore diameter were found to be the most influential parameters. The proposed method can be used to optimize nanoporous coated surfaces for a variety of working fluids.

### 1.3. Laser-Textured Surfaces for Boiling Enhancement

Laser texturing is an increasingly popular method for surface engineering in many fields, including tribology [29] and droplet size separation [30]. As recent references and reviews [31] clearly indicate, laser texturing is also commonly used to functionalize boiling surfaces to enhance their heat transfer performance. This subsection reviews the most influential previous research on the use of direct laser texturing to produce functionalized surfaces for enhanced boiling heat transfer.

A series of studies by Kruse et al., Costa-Greger et al. and Gogos et al. [32,33,34,35,36] focused on femtosecond laser texturing of surfaces to enhance boiling heat transfer. They achieved an enhancement of both the HTC and the CHF on stainless steel and copper surface. More importantly they also showed that a porous oxide/nanoparticle layer formed during the laser texturing process can have significant negative effects on the boiling performance by increasing the thermal resistance of the surface. Kong et al. [37] used direct laser writing to produce laser-induced graphene on polyamide surfaces. When tested with the FC-72 dielectric fluid, a twofold-to-threefold increase in the CHF and HTC was obtained compared to an untreated surface. Eid et al. [38] used laser machining to create cavities with diameters of several hundred micrometers on brass surfaces. The latter were then tested under pool boiling conditions with an alumina nanofluid and the results showed that the major enhancement of heat transfer performance in terms of increased CHF and HTC ensues due to laser-fabricated surface features, while the nanofluid provides a minor additional enhancement. Another series of interconnected studies was performed by Zupančič et al., Gregorčič et al., Može at al., Sitar et al., Zakšek et al. and Voglar et al. [6,39,40,41,42,43,44,45,46,47]. These studies showed that direct laser texturing can be used to create microcavities on boiling surfaces, which are suitable for vapor entrapment and serve as active nucleation sites [39,40]. Furthermore, it was shown that laser-textured surfaces exhibit more favorable stability during long-term exposure to boiling [41], but changes in surface chemistry and morphology appear during their repeated exposure to CHF [42]. Combining the laser texturing with subsequent hydrophobization [6] and/or creating biphilic surfaces with mixed wettability [43,44] was shown to significantly enhance the boiling performance with water and mixtures [45]. Control over the location of nucleating areas through the use of local laser texturing also enabled research of boiling heat transfer mechanisms by utilizing the heat flux partitioning approach [46]. In addition to applying laser texturing to metals and alloys such as copper, aluminum and stainless steel, it was also shown that boiling on silicon surfaces can be significantly enhanced [47]. The latter was also researched by Serdyukov et al. [48], who reported major enhancements of the heat transfer coefficient alongside earlier ONB in comparison with both the polished and the untreated sample. Novel methods of laser surface texturing, such as fabrication of curved, meandering grooves with submicrometer spacing by laser irradiation in liquid [49], could also possibly be applied to functionalize surfaces for enhanced phase-change heat transfer.

### 1.4. Motivation and Goals of the Present Study

The present study aims to further investigate the possibility of significant HTC and CHF enhancements by combining direct laser texturing of copper surfaces with subsequent hydrophobization. Allred et al. [5] have shown that degassed microstructure boiling surfaces that otherwise exhibit superhydrophobicity can be used for efficient boiling heat transfer if boiling is initiated from the Wenzel wetting regime. Our previous study [6], which included fabricated microcavities on aluminum surfaces coated with a nanoscopic layer of a fluorinated silane, further explored and confirmed this idea.

In this study, three types of laser-textured copper surfaces were fabricated and tested both in the as-prepared superhydrophilic state and after hydrophobization with the use of a self-assembled monolayer of fluorinated silane (i.e., in a superhydrophobic state). Three approaches to surface structuring were evaluated and compared to one another, namely fabrication of (i) a crosshatch structure, (ii) shallow channels or (iii) deep channels. The properties of surfaces were evaluated using scanning electron microscopy and contact angle measurements performed before and after boiling experiments. Pool boiling performance of all surfaces was evaluated under atmospheric pressure and at saturated conditions using twice-distilled water. Stability of functionalized surfaces was evaluated through three consecutive experimental runs, characterizing the boiling curve up to CHF incipience. Heat transfer coefficients and critical heat flux values were compared, while a high-speed video analysis was used to analyze bubble dynamics and their departure diameters.

## 2. Materials and Methods

### 2.1. Samples

Samples used within the study were manufactured from a copper rod of electrolytic purity (>99.9% Cu). All samples were sanded with progressively finer sandpaper and finished with a P1200 and P2000 grit paper. Roughness of samples after the pretreatment was approx. *R*_a_ = 0.20 μm as measured with a profilometer. All samples were cleaned with isopropanol to remove dust, grease and other contaminants. One sample was tested without further treatment and used as a reference; it is denoted as the “REF” sample. All other samples underwent laser texturing as described in the following subsection.

### 2.2. Surface Laser Texturing

A nanosecond fiber laser texturing system (JPT Opto-electronics Co., Ltd., M7 30 W MOPA; *λ* = 1064 nm) was used to modify the surface structure of samples. The laser beam is directed across the surface with an F-Theta lens with a working area of 70 × 70 mm^2^ and a focal distance of 100 mm. The focused beam spot diameter is approx. 25 μm, the laser beam quality M^2^ < 1.3 (manufacturer data) and the maximal power of the laser source 30 W. The software ezCAD 2 (Beijing JCZ Technology Co., Ltd., Beijing, China) was used to design the surface treatment patterns and control the laser system during the laser treatment. 

Parameters of the laser system used in the fabrication of each sample were determined through preliminary testing to achieve the desired surface structures. All parameters are provided in Table 1. The first sample was textured with a crosshatch pattern with constant spacing of 60 μm between the consecutive lines. The sample was denoted as “CH”. The second sample was textured only in one direction to create parallel channels with a variable spacing between them. The spacing was cyclically varied between 35 μm and 65 μm by 5 μm to produce porous ridges of various widths and provide the best probability of microcavity formation. This concept was successfully demonstrated and described in detail in our previous publications [6,39,42]. The fluence of laser pulses was reduced to create shallow channels on the sample surface and the sample was accordingly denoted as “SC”. Finally, the third sample was laser textured using the same pattern as the “SC” sample but with a significantly higher pulse fluence to produce deep channels; the sample was denoted as “DC”.

### 2.3. Surface Hydrophobization

Selected samples were hydrophobized using chemical vapor deposition (CVD) of a fluorinated silane. To perform the treatment, 0.05 mL of (heptadecafluoro-1,1,2,2-tetrahydrodecyl)trimethoxysilane (abbreviated to HTMS; Gelest Inc., Morrisville, PA, USA) was mixed with 0.95 mL of toluene (≥99.7%, Honeywell International Inc., Charlotte, NC, USA) in a 5 mL glass vial at room temperature. The samples and the vial were placed in a plastic container, which was then covered with aluminum foil. The container was placed in a preheated oven at 90 °C for 90 min at atmospheric pressure for the self-assembled monolayer to form. After 90 min, the oven was turned off and the container was allowed to cool down before the samples were removed. During the CVD process, a self-assembled monolayer of fluorinated silane molecules is formed on the surface with a thickness of approx. 2.5 nm [50]. This coating process was previously used to functionalize aluminum samples and shown to be sufficiently stable for boiling experiments [6,19,44].

Table 2 lists all samples used in the study together with the observed contact angle of water on their surface as recorded immediately after the last step of their fabrication. Each functionalized sample was produced in two variants: one was only laser textured (no suffix), while the other was hydrophobized after the laser texturing (“-H” suffix). After hydrophobization, all samples exhibited superhydrophobicity with a roll-off angle below 5° and a contact angle higher than 150°. Conversely, all non-hydrophobized laser-textured samples exhibited contact angle below 1° and were in the saturated Wenzel regime [51]. A hydrophobized version of the untreated sample was not fabricated. It was previously shown that hydrophobization of a nominally flat surface only has a limited effect on boiling performance [6], hence this experiment was not repeated.

### 2.4. Surface Analysis

The surface of the samples was analyzed using a scanning electron microscope (SEM; Quattro S, ThermoFisher Scientific Inc., Waltham, MA, USA), utilizing both BSE and SE imaging at an accelerating voltage of 10 kV.

Wettability of the samples was evaluated through sessile drop contact angle measurements with a contact angle goniometer (Ossila Ltd., Sheffield, UK). On each surface, 5 drops (approx. 20 μL) were deposited onto different parts of it and proprietary Ossila software was used to evaluate the resulting images of the contact angle between the water droplet and the surface. All measurements were performed at room temperature.

**Figure 1:**
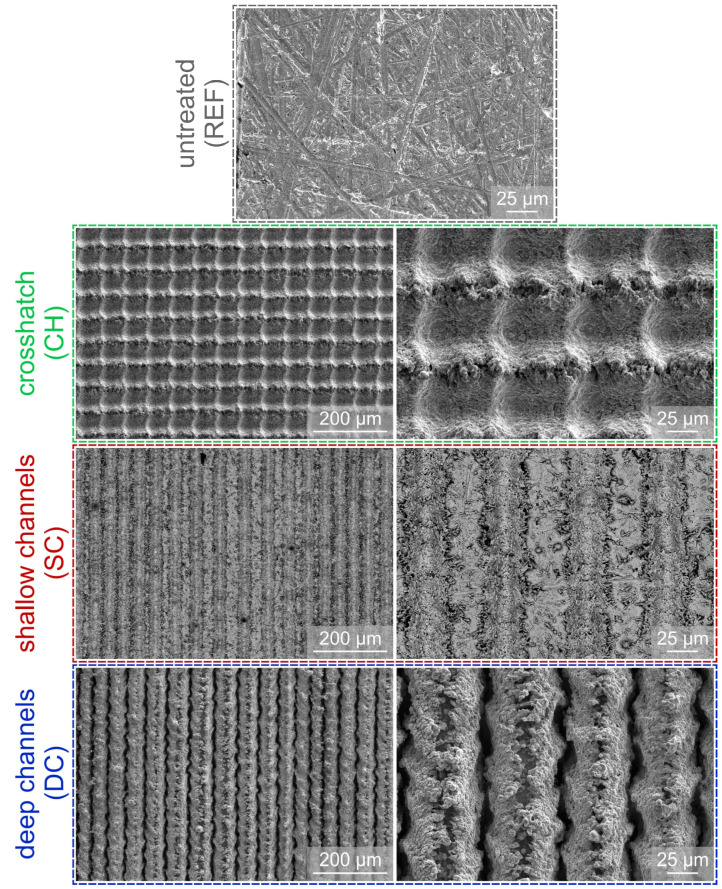
SEM images of the untreated reference surface (REF) and three types of laser-textured surfaces: crosshatch surface (CH), shallow channel surface (SC) and deep channel surface (DC).

Figure 1 shows SEM images of all types of surfaces used within the study at different magnifications. On the untreated reference surface REF, only toolmarks from the sandpaper pretreatment are evident. Crosshatch (CH) sample exhibits a distinct cross surface texture, resulting from the treatment in two directions, perpendicular to one another. The direction of the second laser beam pass is more pronounced as that of the first pass due to the relatively high pulse fluence of the laser treatment. The shallow channel (SC) sample exhibits traces of laser texturing which are approximately as wide as the diameter of the laser beam in the focal position. These channels are normally poorly visible and are highlighted through the use of BSE imaging (SE images are shown for all other surfaces). Finally, the bottom row of Figure 1 shows the morphology of the deep channel (DC) sample, where much deeper traces of the laser beam passes are evident and caused by higher pulse fluence. Both the valley and the ridges produced through solidification of molten and ejected material appear porous with cavities in the micron and submicron range. According to the established literature, such cavities are highly suitable for vapor entrapment and typically provide a major enhancement to boiling performance [6,52,53,54]. 

### 2.5. Pool Boiling Experimental Setup

**Figure 2:**
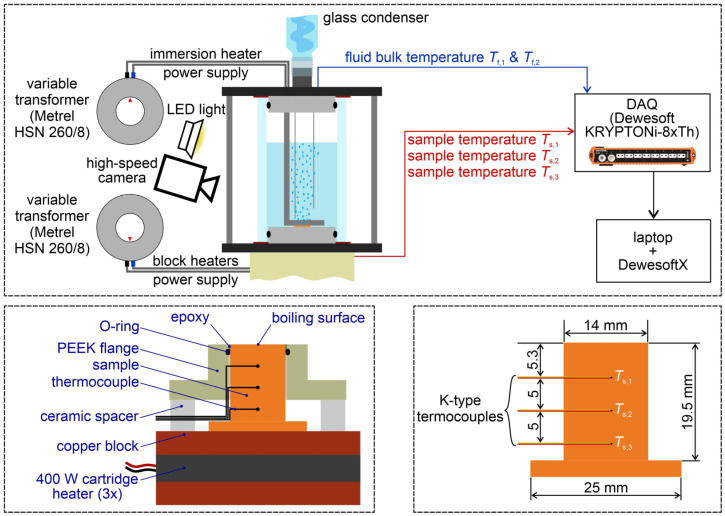
Experimental setup (**top**), heating block and sample assembly (**bottom left**) and sample dimensions including thermocouple locations (**bottom right**).

An evaluation of the pool boiling heat transfer performance was performed with a previously described setup [19,23], hence only a brief description will be provided here. The setup consists of a glass cylinder clamped between two flanges serving as the boiling vessel. The vapor is led through a vent in the upper flange into a glass condenser, from which it is returned to the vessel. The vessel temperature is mounted though two K-type immersion thermocouples, while an immersion heater is used to control the bulk temperature and to degas the fluid before the experiments. The setup is schematically shown in the upper part of Figure 2 alongside the auxiliary equipment for power supply and data acquisition.

The sample is mounted onto a copper heating block, which hosts three 400 W cartridge heaters to supply heat to the bottom of the sample. A PEEK flange is used together with silicone O-rings to provide a seal against the lower flange, through which the entire heater assembly is inserted into the boiling vessel. The sample/heater assembly is shown in the lower left part of Figure 2.

The sample itself is cylindrical with the diameter of the boiling surface being 14 mm. The bottom part of the sample is a flange through which the sample is bolted onto the heater block. Three K-type thermocouples were glued into the sample to record the axial temperature gradient within the sample, which is subsequently used to determine the heat flux and extrapolate the surface temperature. The cross-section of the sample is shown in the bottom right part of Figure 2.

### 2.6. Data Reduction and Measurement Uncertainty

Temperatures within the sample and in the boiling vessel recorded during the measurements were used to calculate the relevant heat transfer parameters. Firstly, the spatial temperature gradient within the sample in the direction of heat conduction was evaluated using the following equation [55]:

(1) $$\frac{\Delta T}{\Delta x}=\frac{T_{s,3}-T_{s,1}}{2\Delta x_{1}}\;$$

where *T*_s,1_ and *T*_s,3_ are temperature measured in the sample in positions denoted in Figure 2, while Δ*x*_1_ = 5 mm represents the distance between two neighboring thermocouples. To calculate the heat flux, thermal conductivity of the copper needs to be accounted for. It was recently shown [55] that a temperature-dependent value should be used to minimize the uncertainty. Hence, a previously defined equation, formulated on the basis of thermal diffusivity measurements, was utilized to evaluate the thermal conductivity of the sample at its mean temperature (calculated as the arithmetic mean of all three temperatures measured in the sample):

(2) $$k\left(T\right)=0.000283T^{2}-0.1646T+378.07$$

The latter equation accepts temperature in °C and returns thermal conductivity in W m^−1^ K^−1^. From the spatial temperature gradient and thermal conductivity, the heat flux was calculated as:

(3) $$\dot{q}=k\frac{\Delta T}{\Delta x}$$

Surface superheat was determined through extrapolation of the surface temperature:

(4) $$T_{w}=T_{s,1}-\frac{\dot{q}\Delta x_{2}}{k}$$

In Equation (4), *T*_w_ denotes temperature of the boiling surface and Δ*x*_2_ = 5.3 mm the distance from the uppermost thermocouple to the surface of the sample. The surface temperature is extrapolated in two calculation steps. First, an estimated value is calculated by using the thermal conductivity evaluated through Equation (2) at temperature *T*_s,1_. In the second step, arithmetic mean temperature between *T*_s,1_ and the estimated *T*_w_ is used to re-evaluate the thermal conductivity and calculate the final boiling surface temperature using Equation (4). Surface superheat is then determined by subtracting the average bulk temperature within the boiling vessel from the calculated surface temperature:

(5) $$T_{w}-T_{\mathrm{sat}}=T_{w}-{\bar{T}}_{f}$$

Finally, the heat transfer coefficient (HTC) is calculated as the ratio between the heat flux and the corresponding surface superheat:

(6) $$h=\frac{\dot{q}}{T_{w}-T_{\mathrm{sat}}}$$

Methodology suggested in [55] was used to evaluate the measurement uncertainty of all three main heat transfer parameters. Contributing uncertainties of individual parameters include (i) the uncertainty of the distance between the thermocouples (*u*(Δ*x*_1_) = 0.16 mm; evaluated experimentally), (ii) the uncertainty of the distance between the uppermost thermocouple and the boiling surface (*u*(Δ*x*_2_) = 0.18 mm; evaluated experimentally), (iii) the uncertainty of the thermocouple temperature measurements (*u*(*T*) = 0.19 K at 100 °C and *u*(*T*) = 0.30 K at 250 °C; evaluated experimentally) and (iv) the uncertainty of thermal conductivity (*u*_r_(*k*) ≅ 1.5%; estimated based on the accuracy of thermal diffusivity measurements).

Measurement uncertainty was evaluated on three selected samples at heat flux values of approx. 250 kW m^−2^ and at 1000 kW m^−2^, representing low and high heat flux within the nucleate boiling regime. The heat flux uncertainty on the untreated reference sample is 4.3% at 250 kW m^−2^ and 2.4% at 1000 kW m^−2^. These uncertainty values are also valid for other samples since heat flux uncertainty is not influenced by the heat transfer performance of an individual sample. The surface superheat uncertainty on the untreated reference sample is 2.3% at 250 kW m^−2^ and 2.5% at 1000 kW m^−2^. The heat transfer coefficient uncertainty on the untreated reference sample is 4.8% at 250 kW m^−2^ and 3.5% at 1000 kW m^−2^. The second selected sample for the uncertainty evaluation was the SC-H sample, which exhibited the highest overall CHF values. On this sample, the surface superheat uncertainty is 4.3% at 250 kW m^−2^ and 4.9% at 1000 kW m^−2^, while the heat transfer coefficient uncertainty was calculated to be 6.1% at 250 kW m^−2^ and 5.3% at 1000 kW m^−2^. Finally, measurement uncertainty evaluation was also performed for the DC-H sample, which exhibited the highest heat transfer coefficient values and by far the lowest surface superheat values. On this sample, the surface superheat uncertainty is 7.2% at 250 kW m^−2^ and 14.5% at 1000 kW m^−2^, while the heat transfer coefficient uncertainty was calculated to be 8.4% at 250 kW m^−2^ and 14.7% at 1000 kW m^−2^. Higher uncertainty values are a consequence of very low surface superheat values.

### 2.7. Measurement Protocol

Identical measurement protocol was used to determine the heat transfer performance of all samples. After the surface of the sample was functionalized, the sample was mounted onto the heater assembly and the edge was sealed with epoxy to prevent parasitic nucleation. The assembly was then mounted through the bottom flange of the boiling vessel, which was subsequently filled with approx. 200 mL of twice-distilled water. The water was heated to saturation and degassed through vigorous boiling for at least 45 min prior to first measurements. During this time, a heat flux of approx. 400 kW m^−2^ was applied to the sample to establish nucleate boiling and help get rid of air, which was possibly entrapped on the surface.

Before starting an experimental run, the sample and the water were cooled to 90 °C to condense any entrapped vapor. The temperature of the water was then brought to saturation again, while the heating power on the cartridge heaters below the sample was slowly increased. During the measurement, the heating power was very slowly but continuously increased at a rate of approx. 0.2 kW m^−2^ s^−1^ within the natural convection regime and up to 2 kW m^−2^ s^−1^ within the nucleate boiling regime. This allows for faster evaluation of the boiling curve and prevents possible changes in the boiling surface due to prolonged boiling and/or exposure to water from influencing the results. This methodology was experimentally and numerically confirmed to be a very good approximation of the steady-state measurements [6,19].

The measurement was conducted until the CHF was reached. At this point, the heaters were turned off and the sample was allowed to cool down below saturation temperature of the water. Afterwards, the measurement was repeated until a total of three runs were conducted on each sample.

During the measurements, a high-speed camera (Photron FASTCAM Mini UX100, Photron Limited, Tokyo, Japan) was used to capture the phenomena on and above the boiling surface. Recordings were made at heat fluxes of 50 kW m^−2^ and 100 kW m^−2^, where individual bubbles were still observable. At higher heat flux values, the intense coalescence of bubbles on most functionalized surfaces obscured the dynamics and identification of diameters of individual bubbles.

## 3. Results

### 3.1. Reference Experiments

**Figure 3:**
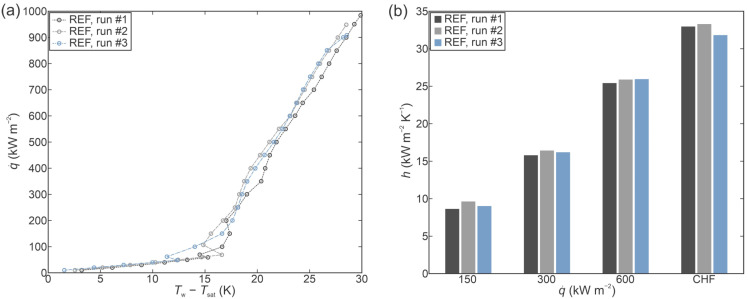
Boiling performance of the untreated reference sample (REF) evaluated through (**a**) boiling curves and (**b**) heat transfer coefficients at selected heat fluxes.

Firstly, boiling experiments were conducted on an untreated surface with the intention of setting a baseline for further experiments with functionalized surfaces. Comparison of boiling curves obtained during three consecutive measurements on the REF surface are shown in Figure 3a. The obtained critical heat flux of approx. 1000 kW m^−2^ matches typical values reported in the literature [55] for untreated copper surfaces operating under the given test conditions (saturated boiling of water at atmospheric pressure). Boiling curves remained stable and within the measurement uncertainty (temperature-wise) during the three repetitions of the test. A small decrease in CHF was observed and could be attributed to the appearance of different surface oxide species due to high temperatures of the sample when the CHF incipience occurs. This was previously reported in [42], where transformation of CuO into Cu_2_O was shown to take place during the post-CHF temperature increase as a result of the low-temperature annealing effect.

Heat transfer coefficients recorded on the reference surface are shown in Figure 3b. The highest values are reached at the point of CHF incipience with the maximal recorded value of 33.3 kW m^−2^ K^−1^ being observed during the second experimental run. In comparison to one another, the heat transfer coefficient values measured during the three consecutive runs generally differ by less than 10%.

### 3.2. Performance of Individual Functionalized Surfaces

Performance of functionalized surfaces was evaluated using the same methodology as for the untreated reference sample. For each type of laser-engineered surface morphology, results for the as-fabricated (superhydrophilic) and hydrophobized (superhydrophobic) version of the surface are shown and compared.

**Figure 4:**
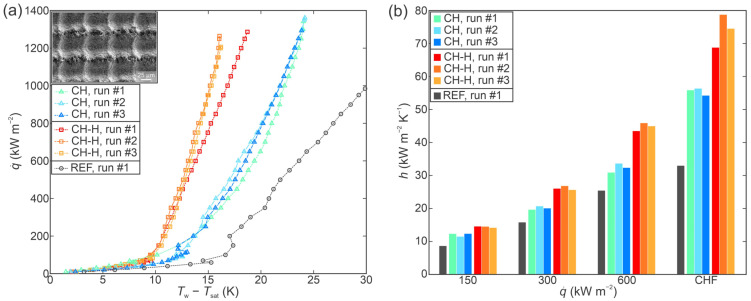
Boiling performance of the crosshatch sample (CH) evaluated through (**a**) boiling curves and (**b**) heat transfer coefficients at selected heat fluxes.

Figure 4a shows a comparison of boiling curves recorded on the crosshatch patterned surface in the as-prepared state (CH) and hydrophobized state (CH-H). Enhanced CHF values in comparison with the untreated surface (shown with a grey curve) were obtained in both cases. Specifically, an enhancement of 36% and 31% was recorded during the first experimental run on surfaces CH and CH-H in comparison with the first run on the untreated surface, respectively. Positive boiling curve stability was observed for the hydrophilic CH sample, while a slight decrease in surface superheat (approx. 2.5 K at CHF incipience) was observed during the second and third experimental run in comparison with the first test on surface CH-H. Since this shift is greater than the surface superheat measurement uncertainty, it needs to be attributed to surface changes. The most likely explanation is additional removal of air entrapped on the surface during the first CHF incipience or changes in the surface chemistry and/or morphology.

Figure 4b shows a comparison of heat transfer coefficients at specific heat flux levels for the CH, CH-H and the reference surface. On both functionalized surfaces, the highest HTC values were again recorded at the point of CHF incipience with the highest value for the as-prepared CH surface being 56.3 kW m^−2^ K^−1^ and for the hydrophobized CH-H surface being 78.7 kW m^−2^ K^−1^. The latter values represent an improvement of 69% and 136% in comparison with the maximal value recorded on the untreated surface. For both versions of the crosshatch surface, critical heat flux values are within 10% of each other.

**Figure 5:**
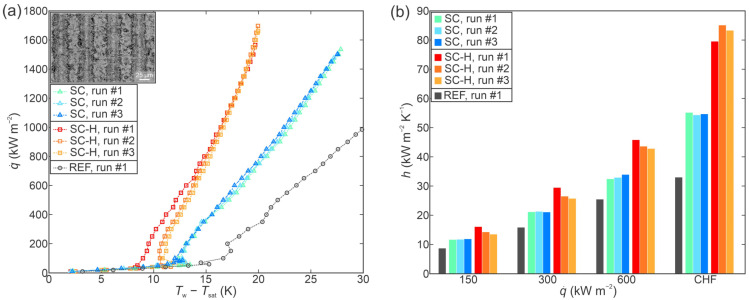
Boiling performance of the shallow channel sample (SC) evaluated through (**a**) boiling curves and (**b**) heat transfer coefficients at selected heat fluxes.

Figure 5a shows a comparison of boiling curves obtained on the as-prepared surface with shallow channels (SC) and its hydrophobized variant (SC-H) alongside the boiling curve for the untreated reference surface. Both functionalized surfaces exhibit significantly enhanced CHF values with a 56% and a 59% enhancement recorded on the SC and the SC-H surface, respectively, in comparison with the untreated surface. 

The highest CHF value of 1697 kW m^−2^ was recorded during the third experimental run on the SC-H surface, where the CHF value gradually increased during the measurements by approx. 8% from the first to the last measurement. Otherwise, very positive stability was observed on both the SC and SC-H surface with minute deviations between the repeated runs on each surface.

Heat transfer coefficients recorded on the SC and SC-H surfaces are compared to one another and to those for the untreated sample in Figure 5b. Both functionalized surfaces exhibited increased HTC values with the highest values of 55.1 kW m^−2^ K^−1^ and 85.1 kW m^−2^ K^−1^ recorded at the point of CHF incipience on the SC and SC-H surface, respectively. This represents a 65% and a 156% enhancement over the performance of the untreated surface, respectively. Favorable stability of the heat transfer coefficient was also confirmed with stable values recorded at the same heat flux levels during consecutive tests on each surface.

**Figure 6:**
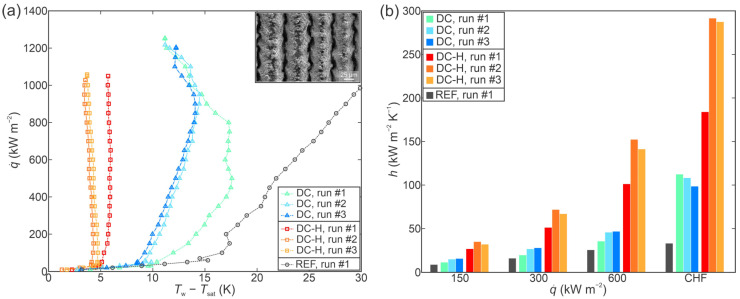
Boiling performance of the deep channel sample (DC) evaluated through (**a**) boiling curves and (**b**) heat transfer coefficients at selected heat fluxes.

Finally, Figure 6a compares the boiling curves recorded on the surface with deep channels in the as-fabricated hydrophilic state (DC) and the hydrophobized state (DC-H), while the boiling curve for the untreated surface is again shown in grey color. Both version of the functionalized surface achieved greater CHF values in comparison with the reference surface, but the enhancement was not as pronounced as on other types of functionalized surfaces (i.e., crosshatch and shallow channel surfaces). Specifically, a CHF of 1253 kW m^−2^ was recorded during the first run on the DC surface, marking a 27% enhancement over the reference surface. A CHF of 1050 kW m^−2^ was recorded during the first run on the hydrophobized DC-H surface, marking an enhancement of 7% over the performance of the untreated surface. The CHF values recording during three consecutive evaluations of an individual surface remained very stable.

While the CHF enhancement achieved using the deep channel surfaces was limited, these surfaces provided by far the greatest heat transfer coefficients recorded within the study. A comparison of HTC values at selected heat flux levels is shown in Figure 6b. On the hydrophilic DC surface, a highest HTC of 112.3 kW m^−2^ K^−1^ was recorded at CHF incipience during the first experimental run, representing a 237% enhancement over the performance of the untreated surface. Exceptional heat transfer performance was also observed on the hydrophobized variant of the deep channel surface (i.e., DC-H), where the highest recorded heat transfer coefficient measured 291.4 kW m^−2^ K^−1^ and was recorded at the point of CHF incipience during the second experimental run. This HTC value is one order of magnitude greater than that exhibited by the untreated surface, marking a 775% enhancement.

While the boiling curves exhibited favorable stability in terms of CHF values, a notable shift towards lower surface superheat values was detected after the first boiling run. The maximal shift measured approx. 5 K on the DC surface (observed at 500 kW m^−2^) and approx. 2 K on the DC-H surface (observed at CHF incipience). However, once the shift had occurred, the position of the boiling curve stabilized, which was previously observed [42] on laser-textured copper surfaces during multiple consecutive boiling tests.

### 3.3. Comparison of Boiling Performance

**Figure 7:**
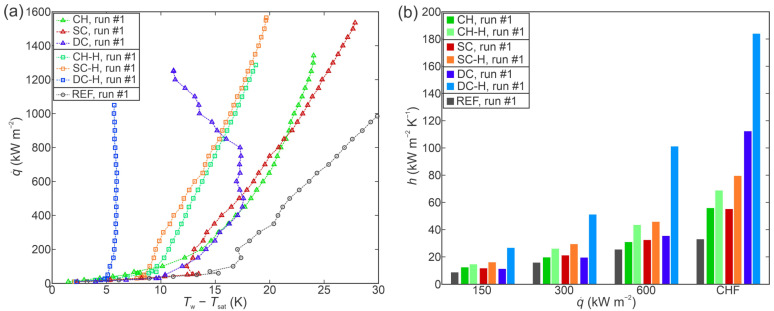
Comparison of boiling performance of all samples during the first experimental run through (**a**) boiling curves and (**b**) heat transfer coefficients at selected heat fluxes.

To compare the heat transfer performance of the functionalized surfaces to one another, two figures are presented. Firstly, Figure 7a shows a comparison of the boiling curves obtained on all surfaces during the first experimental run, while Figure 7b shows a comparison of the corresponding heat transfer coefficients. For all three types of functionalized surfaces, the following general findings can be made: (i) the hydrophobized variant of the surface always outperformed the hydrophilic variant in terms of the heat transfer coefficient achieved at the same heat flux and (ii) the hydrophilic variant of each surface always exhibited either a comparable or higher CHF value than its hydrophobic counterpart. All surfaces exhibited enhanced HTC and CHF values in comparison with the untreated surface.

**Figure 8:**
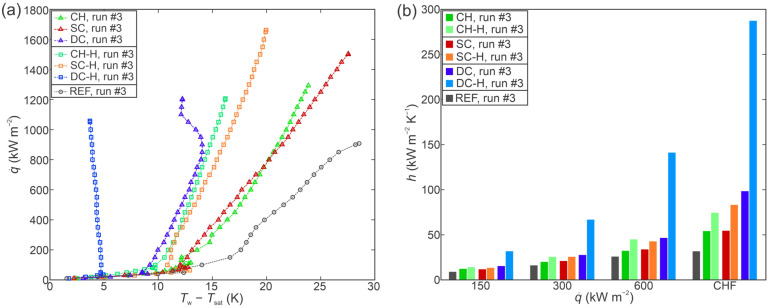
Comparison of boiling performance of all samples during the third experimental run through (**a**) boiling curves and (**b**) heat transfer coefficients at selected heat fluxes.

Since a shift in the boiling curve during consecutive boiling tests was detected on some functionalized surfaces, a further comparison of their performance is made in Figure 8a in terms of boiling curves and Figure 8b in terms of heat transfer coefficient, in both cases recorded during the last (i.e., third) experimental run. For all three types of functionalized surfaces, the hydrophobized variant also outperformed its hydrophilic counterpart in terms of HTC at every heat flux level. However, a gradual increase in the CHF value was observed on the hydrophobic version of the shallow channel surface, meaning that the hydrophilic surfaces no longer universally outperformed the hydrophobic surfaces in terms of the CHF value. Since a positive shift in the boiling curve (i.e., towards lower surface superheat values) was observed (if observed at all), the enhancement provided by the functionalized surfaces in comparison with the untreated reference surface mostly increased during the second and third experimental run.

### 3.4. High-Speed Videography Analysis

**Figure 9:**
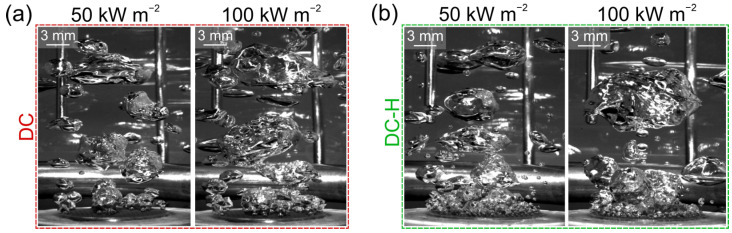
High-speed snapshots of the boiling process at 50 kW m^−2^ and 100 kW m^−2^ on (**a**) hydrophilic and (**b**) hydrophobic version of the deep channel surface (DC and DC-H, respectively).

To help explain the differences in boiling heat transfer performance both between the three types of surfaces and between the hydrophilic and hydrophobic variant of each surface, high-speed videography results were analyzed to determine the bubble diameter on each surface at two heat flux levels, where individual bubbles could still be observed. An example of the snapshots of the boiling process is shown in Figure 9, where bubble dynamics on the hydrophilic deep channel (DC) surface at 50 kW m^−2^ and 100 kW m^−2^ (Figure 9a) are compared to the dynamics on the hydrophobic deep channel surface (DC-H) at the same heat flux levels (Figure 9b). It is clearly observable that the hydrophobized variant of the surface provides significantly enhanced active nucleation site density and smaller vapor bubbles were formed during the boiling process.

At least 10 bubbles were analyzed during boiling on each surface at each heat flux level used in the analysis to determine the average bubble diameter at the moment of departure (i.e., the bubble departure diameter) and the standard deviation of the values. These results are summarized in Table 3, from which it is observable that hydrophobized variants of each surface (with the exception of SC-H at 50 kW m^−2^) exhibited significantly smaller bubble departure diameters than the hydrophilic counterpart at the same heat flux.

## 4. Discussions

The results of experimental evaluation of boiling heat transfer of functionalized surfaces have shown that all fabricated surfaces are capable of enhancing both the critical heat flux and the heat transfer coefficient in comparison with the untreated reference surfaces. Here, we aim to explain the mechanisms behind the enhancement and interpret the reasons for differences between different surface morphologies (i.e., the three types of surfaces—CH, SC and DC) and between the hydrophobic and hydrophilic variant of an individual surface (i.e., surfaces with or without the “-H” suffix).

Laser texturing modifies the surface morphology and surface chemistry of the treated surface. Typically, laser ablation as used within this study (and most others) causes the appearance of either singular craters or lines of craters, which form valleys surrounded by ridges of resolidified material. The latter is typically rich in oxide species and porous to some extent. When the line spacing between consecutive laser beam passes is appropriately matched with the width of laser-induced damage to the surface, microcavities appear on the ridges. These were previously shown [6,39,40] to significantly enhance the boiling performance by serving as active nucleation sites from which vapor bubbles form and grow. Such microcavities are observable on the SEM micrographs of surfaces CH and DC as shown in Figure 2. Additionally, microcavities can also appear without the overlap of adjacent ridges [40], which is the case for surface SC in Figure 2. Presence of microcavities enables vapor embryos to become entrapped on the surface and start nucleating at low surface superheat values. The superior performance of laser-textured surfaces fabricated within this study can therefore be partly contributed to the morphology of laser-textured structures on the functionalized surfaces.

Various oxide species tend to form during laser texturing of metallic materials when the ablated and/or molten material is exposed to the ambient atmosphere containing oxygen. In case of copper, the predominant oxide species will be the copper(II) oxide CuO, while the copper(I) oxide (Cu_2_O) can also be present and will be prevalent if the laser texturing is performed in an inert atmosphere such as argon [42]. Universally, laser-textured surfaces tend to exhibit (super)hydrophilicity immediately after laser texturing is carried out, since the oxide species boast high surface energy. However, after exposure to ambient air, the wettability of laser-textured surfaces decreases [56], which can be attributed to the deposition of various intrinsically hydrophobic compounds onto the surface [57,58,59]. All surfaces tested within the study in the as-prepared (i.e., superhydrophilic) state exhibited significantly increased CHF values, which is commonly reported for well-wettable surfaces [23,60,61,62]. Therefore, an assumption could be made that the surface hydrophilicity provided an additional enhancement in the combination with the aforementioned vapor-entrapping microstructure.

However, the hydrophobized variants of all functionalized surfaces also provided a CHF enhancement in comparison with the untreated surface. While poorly wettable (i.e., hydrophobic and superhydrophobic) surfaces have historically been dismissed as suitable for boiling enhancement, it was shown that with appropriate degassing, the Wenzel wetting regime can be achieved under boiling conditions without the negative effects of air/vapor entrapment across the entire surface [5] that would lead to an early transition to film boiling. When superhydrophobicity is combined with microcavity surface microstructure, significant enhancements of boiling performance have been obtained on aluminum samples [6,19]. Here, a similar result was obtained on hydrophobized laser-textured copper surfaces with increased CHF and HTC values recorded on all three types of hydrophobized surfaces. Since similar CHF values were obtained for each pair of surfaces with the same microstructure as evident from Figure 7 and Figure 8, it can be concluded that surface hydrophilicity has a much smaller influence on the CHF than the appropriate surface microstructure.

In addition to variations in the surface microstructure, the oxide layer thickness can also significantly influence boiling performance. It was convincingly shown in multiple studies by Kruse et al. [33,34,36] that the thick oxide layers with low thermal conductivity can degrade boiling performance. In the present study, crosshatch and deep channel (CH and DC) surfaces were textured at much higher laser pulse fluence values, resulting in greater laser-induced damage to the surface and thicker oxide layers [63,64]. Conversely, a lower pulse fluence was used on the shallow channel (SC) surface, resulting in a thinner oxide layer (i.e., less thermal energy per unit area was introduced to the sample during the laser texturing) and less vapor trapping structure. Deep structures present on CH and DC surfaces, which greatly enhance the HTC and ONB, become problematic at high heat flux levels due to vapor spreading and a possible local dryout, which can cause an early CHF onset. This matches experimental observations in terms of CHF values very well, as the highest CHF value by far was recorded on both versions of the SC surface.

Surface hydrophobization was found to shift the boiling curve towards lower surface superheat values on all samples used within the study. Lower surface energy provided by the fluorinated monolayer coating positively influences nucleation characteristics by allowing for easier vapor entrapment and onset of nucleate boiling at lower surface superheat values [6]. When this is combined with vapor entrapping microstructure such as microcavities, the bubble diameter is decreased significantly in comparison with the hydrophilic counterparts, as evident from the values in Table 3 for both the CH-H and DC-H surfaces. This matches previous reports of reduced bubble diameters by 50% or more on hydrophobized microcavity surfaces [19].

Finally, a recent study [15] provided an in-depth understanding of how the surface morphology and wettability affect boiling heat transfer. Specifically, experiments were first performed on a hydrophilic surface, which was subsequently hydrophobized and tested again in a hydrophobic state, so that the exact same surface morphology was present during both tests. High-speed recording in the visible and IR spectrum revealed that the smaller bubble diameter and the larger density of active nucleation sites positively affect the uniformity of the boiling surface temperature. It can therefore be reasoned that the smaller bubble departure diameters as observed within the present study on hydrophobized surfaces also correspond to narrower surface temperature distributions and contribute to the extremely high HTC values observed on the surface with the smallest bubbles, i.e., the hydrophobized deep channel surface (DC-H).

## 5. Conclusions

Three types of laser-textured surfaces were fabricated and tested both in the as-fabricated superhydrophilic state and superhydrophobic state after hydrophobization with a self-assembled monolayer of a fluorinated silane. Each surface was evaluated under saturated pool boiling conditions at atmospheric pressure using water as the working fluid. The following conclusions are made based on the obtained results:

1. All laser-functionalized surfaces exhibited enhanced boiling heat transfer performance in comparison with an untreated reference surface. The recorded highest critical heat flux enhancement was achieved on a hydrophobized shallow channel surface (SC-H), where a CHF of 1697 kW m^−2^ was recorded during the third experimental run.
2. In addition to increased critical heat flux, significant enhancements of the heat transfer coefficient were observed. Specifically, the hydrophobized surface with deep channels (DC-H) provided an HTC of 291.4 kW m^−2^ K^−1^ at CHF incipience, which represents a 775% enhancement over the highest values recorded on the untreated reference.
3. Functionalized surfaces exhibited positive stability and repeatable boiling curves. A shift towards lower superheat values was observed on the deep channel surfaces after the first experimental run, which may be attributed to additional degassing of the surface during the post-CHF transition towards film boiling.
4. Surface microstructure was identified as the key reason for enhanced heat transfer parameters. Despite large differences in surface wettability, hydrophobized surfaces exhibited comparable (and in one case even higher) CHF values in comparison with their hydrophilic counterparts, which are traditionally considered as more favorable for achieving high CHF values.
5. A significant reduction in bubble departure diameter was observed on the best-performing surface DC-H, which exhibited the highest HTC value. The small bubble departure diameter is attributed to effective vapor entrapment in the deep surface structures and is pointed out as a major contributing reason for the observed extreme boiling heat transfer performance.

Future research will be focused on pursuing individual factors contributing to higher heat transfer performance as identified within this study by optimizing surface treatment parameters to provide the highest CHF or HTC possible. Additionally, we will attempt to combine both approaches to produce heterogeneously wettable (i.e., biphilic) surfaces to concomitantly exploit the advantage granted by different types of laser-made surface structures.

## Figures and Tables

**Table 1:** Laser-texturing used to functionalize the copper samples.

Sample	Pulse Length (ns)	Pulse Frequency (kHz)	Scanning Speed (mm s^−1^)	Average Fluence (J cm^−2^)	Pattern
CH & CH-H	40	125	125	48.9	crosshatch (0° and 90°), spacing Δ*x* = 60 μm
SC & SC-H	30	500	500	12.2	parallel lines (0°), cyclically variable spacing Δ*x* = {35–65} μm
DC & DC-H	45	110	110	55.6	parallel lines (0°), cyclically variable spacing Δ*x* = {35–65} μm

**Table 2:** List of samples used in the study.

Sample	Treatment	Description	Contact Angle
REF	none	untreated reference sample	93°
CH	laser texturing	hydrophilic sample with a crosshatch pattern	<1°
CH-H	laser texturing + hydrophobization	hydrophobized sample with a crosshatch pattern	160°
SC	laser texturing	hydrophilic sample with a pattern of shallow channels	<1°
SC-H	laser texturing + hydrophobization	hydrophobized sample with a pattern of shallow channels	154°
DC	laser texturing	hydrophilic sample with a pattern of deep channels	<1°
DC-H	laser texturing + hydrophobization	hydrophobized sample with a pattern of deep channels	154°

**Table 3:** Bubble departure diameters at 50 kW m^−2^ and 100 kW m^−2^ including standard deviation.

Sample	*D*_b_ @ 50 kW m^−2^ (mm)	*D*_b_ @ 100 kW m^−2^ (mm)
REF	1.6 ± 0.3	2.4 ± 0.3
CH	2.0 ± 0.3	3.1 ± 0.4
CH-H	1.2 ± 0.1	2.6 ± 0.2
SC	2.0 ± 0.1	2.0 ± 0.3
SC-H	3.1 ± 0.7	1.8 ± 0.2
DC	1.1 ± 0.1	1.6 ± 0.2
DC-H	0.7 ± 0.1	1.0 ± 0.2

## Data Availability

Data is available from the corresponding author upon reasonable request.
